# Seagrass vegetation and meiofauna enhance the bacterial abundance in the Baltic Sea sediments (Puck Bay)

**DOI:** 10.1007/s11356-015-5049-7

**Published:** 2015-07-17

**Authors:** Emilia Jankowska, Katarzyna Jankowska, Maria Włodarska-Kowalczuk

**Affiliations:** Institute of Oceanology Polish Academy of Sciences, Powstańców Warszawy 55, 81-712 Sopot, Poland; Gdańsk University of Technology, Gabriela Narutowicza 11/12, 80-233 Gdańsk, Poland

**Keywords:** Benthic bacteria, Sediment characteristics, Benthic communities, Eelgrass meadows, Baltic Sea

## Abstract

This study presents the first report on bacterial communities in the sediments of eelgrass (*Zostera marina*) meadows in the shallow southern Baltic Sea (Puck Bay). Total bacterial cell numbers (TBNs) and bacteria biomass (BBM) assessed with the use of epifluorescence microscope and Norland’s formula were compared between bare and vegetated sediments at two localities and in two sampling summer months. Significantly higher TBNs and BBM (PERMANOVA tests, *P* < 0.05) were recorded at bottom covered by the seagrass meadows in both localities and in both sampling months. The relationships between bacteria characteristics and environmental factors (grain size, organic matter, photopigments in sediments), meiofauna and macrofauna densities, as well as macrophyte vegetation characteristics (shoot density, phytobenthos biomass) were tested using PERMANOVA distance-based linear model (DISTLM) procedures and showed that the main factors explaining bacteria characteristics are bottom type (vegetated vs. unvegetated) and meiofauna density. These two factors explained together 48.3 % of variability in TBN and 40.5 % in BBM, and their impacts did not overlap (as indicated by DISTLM sequential tests) demonstrating the different natures of these relationships. The effects of seagrass were most probably related to the increase of organic matter and providing habitat while higher numbers of meiofauna organisms may have stimulated the bacterial growth by increased grazing.

Eelgrass plants are important components of marine coastal ecosystems as habitat-forming organisms (Green and Short 2003) and ecosystem engineers (Hemminga and Duarte 2000). Seagrass vegetation can stabilize the sediment, enhance organic matter accumulation, and thus increase the availability of food (Baden et al. 2010) as well as provide a shelter both for adult and juvenile marine organisms. There were a lot of studies indicating higher density and diversity of fauna inhabiting seagrass meadows (Baden and Böstrom 2001; Böstrom and Bonsdorff 1997). However, the vast majority of those studies focus on macrobenthic invertebrates and fish (e.g., Webster et al. 1998; Arrivilliga and Baltz 1999; Bouma et al. 2009; Herkul and Kotta 2009), significantly less on meiofauna (e.g., Daudi et al. 2012; De Troch et al. 2005), and only a few published reports include microorganisms (e.g., Danovaro et al. 1994). Benthic bacteria are important components of the nutrient cycling, play a role of both benthic primary producers and heterotrophic consumers in sedimentary systems of seagrass meadows (Donnelly and Herbert 1999). Some forms of bacterial activity are responsible for nitrogen and phosphorous cycling that is crucial for seagrass productivity and thus survival. Microbes are involved in regeneration of nutrients necessary for seagrass growth (Donnelly and Herbert 1999). On the other hand, microorganisms decompose seagrass tissues into detritus, making them available for consumers. They thus play a significant role in seagrass-derived organic matter flow into the benthic food chain (Oakes et al. 2012). The environmental conditions provided by seagrass meadows (reduced water turbulence, higher concentrations of organic matter, and fine sediments) can enhance the growth of bacteria communities. Previous studies in *Posidonia oceanica* meadows documented strong relationship between the dynamics of the seagrass growth and the development of the microbial community associated with the rhizosphere (García-Martínez et al. 2008). García-Martínez et al. (2008) showed that the decline of the seagrass induced the decline of total bacterial abundance. High biomass of bacterial communities inhabiting sediments of *P. oceanica* meadows in the Ligurian Sea was documented by Danovaro et al. (1994). Most of the studies of microbial components of seagrass meadow systems were performed in warmer zones (The Mediterranean Sea and in Moreton Bay, Australia) and in meadows created respectively by *P. oceanica* or *Zostera capricorni* (Canon et al. 1998; Moriarty and Pollard 1982). No reports on bacteria in temperate meadows created by *Zostera marina* in the Baltic Sea have been hitherto published. The effects of this particular species (that differs in morphology and biomass not only from the *Posidonia* species but also from other *Zostera* species) on microbial standing stocks remain unknown. In general, the southern Baltic Sea meadows remain relatively unexplored. The limited research effort in this specific habitat results from dramatic changes in eelgrass distribution within the last century in this region (Kruk-Dowigałło 1991). *Z. marina* meadows areal distribution dramatically decreased from 80 % to less than 10 % coverage of the Puck Bay area between the 1960s and 1980s (Kruk-Dowigałło 1991). Recently, natural seagrass recovery and rapid increase in the spatial coverage of the eelgrass meadow has been reported (Jankowska et al. 2014). Details of seasonal variability (including the summer months) in macrophyte vegetation traits are described in Jankowska et al. (2014). During that study, total macrophyte biomass was dominated by eelgrass *Z. marina* (50.6 %) and filamentous algae *Pylaiella littoralis* (4.7 %). The shoot density of the seagrass ranged from 180 to 220 shoots m^−2^, while the total biomass of macrophytes associated with eelgrass varied from 30 to 60 g dry wt m^−2^. It remains unexplored if and how this change in habitat distribution (the recent recovery of eelgrass meadows) will change the structure and functioning of the benthic system of the southern Baltic coastal waters, including its bacterial components. Hereby we present the results of a pilot study comparing the basic parameters (abundance and biomass) of bacterial communities in eelgrass vegetated sediments and bare sandy bottom of the shallow Puck Bay (southern Baltic Sea).

Samples were collected by SCUBA diving, in the summer season of 2011, on 1 June and 19 July, when seagrass vegetation is the most developed in terms of shoot density and biomass (Jankowska et al. 2014). Two localities were chosen: (1) in the inner part of the Puck Bay close to the sandbars of Ryf Mew (depth of 3 m) and (2) in the outer part—east of Jastarnia (depth 1.5 m, Fig. 1). At both sampling locations, five replicate sets of samples were taken on vegetated and unvegetated bottoms. A set of samples included a sample of phytobenthos, meiofauna (upper 10 cm), macrofauna (upper 15 cm), sediment samples for bacteria and sediment characteristics (upper 2 cm), grain size, particulate organic carbon (POC) and total nitrogen (TN) concentrations, stable isotopes signatures of δ^13^C, δ^15^N and photosynthetic pigments concentration. Sediment samples for bacteria analysis were collected with a small core (2 cm diameter, upper 2 cm) and stored in sterile plastic tubes in prefiltrated (0.2 μm) formaldehyde (2 % final concentration). The procedures of collection and treatment of macrophytes, macrofauna and meiofauna, and sediment samples are described by Jankowska et al. (2014).

**Fig. 1:**
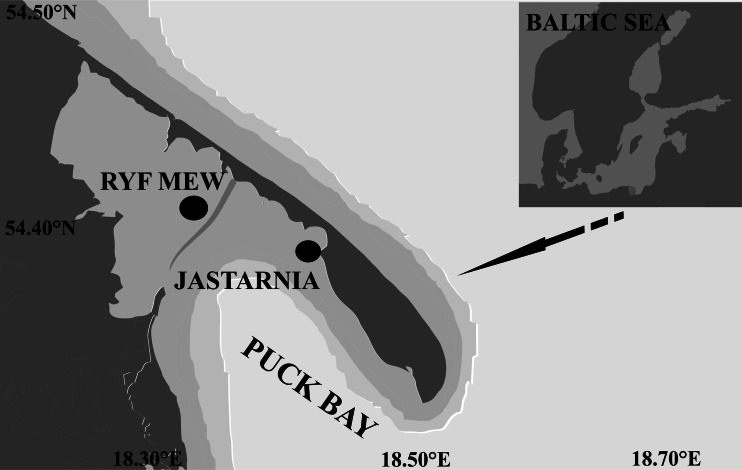
Sampling locations in the Puck Bay (Baltic Sea)

In the laboratory, 2 g of sediments collected for bacteria analyses were mixed with 5 ml of Ringer solution (diluted 1:4 and prefiltered 0.2 μm) and left for 10 min. Samples were sonificated three times (3 × 60 s with 60-s delay, using Sonifier Tansonic Labor 2000), then washed eight times with 5 ml of Ringer solution (Epstein and Rossel 1995, modified). Subsamples of 0.15 ml were taken from the supernatant and mixed with 0.85 ml of Ringer solution, stained for 15 min with 4′,6-diamidino-2-phenylindole (DAPI) [1 μg ml^−1^] and filtered on black nuclepore filters (polycarbonate, 0.2 lam filters, 25 mm diameter) (Porter and Feig 1980). The filters were air dried in the dark and mounted on microscope slides with the use of citifluor (Agar Scientific, England). Bacteria were enumerated with a Nikon epifluorescence microscope (which included monochromatic camera, PC and MultiSkan Bace V.8.08 (CSS) software) under 1200-fold magnification with 100-W mercury lamp, 365-nm excitation filter, 420-nm barrier filter, and 400-nm dichroic mirror. Bacteria cells were counted in 20 microscope fields. The mean value based on the values obtained from the three subsamples was calculated for each sample. The biomass of bacteria was estimated from the mean volume of cells using Norland’s formula (1993). Analysis of counting and size data was performed with Excel macros as described by Świątecki (1997).

Differences in the bacteria univariate characteristics (total bacterial numbers—TBNs, bacterial biomass—BBM) among groups of samples defined by three factors (Mt—month, Lt—locality (station), BT—bottom type) were tested using a three-way PERMANOVA model based on a similarity matrix created on Euclidean distances among samples. Relationships between environmental variables, meiofauna and macrofauna densities, and bacteria abundance and biomass were investigated using the distance-based linear model (DISTLM) procedure in PERMANOVA+ (Anderson et al. 2008). Thirteen variables used for the model included descriptors of organic matter quantity and quality (δ^13^C, δ^15^N, POC, POC/TN), photosynthetic pigments (chl *a*, chloroplastic pigment equivalents (CPE), % chl *a*—percentage of chl *a* in CPE, chl *a*/POC), granulometric characteristics of sediments (mean grain size, sorting, fine sand, and coarse sand fractions), two descriptors of associated zoobenthic community (meiofauna density 10 cm^2^, macrofauna density 0.1 m^2^), two descriptors of seagrass meadow characteristics (shoot density, phytobenthos biomass), and three categorical (nominal) variables (Mt, Lt, BT). Details of seasonal and spatial variability (including the summer months considered in the present study) in meiofauna density and sediment characteristics are reported in Jankowska et al. (2014) and macrofauna density in Włodarska-Kowalczuk et al (2014). During the two summer months, the significant contrasts between the two bottom types were documented for POC, PON, δ^15^N (*P* < 0.001), chlorophyll *a* (*P* < 0.05), and CPE concentration (*P* < 0.01), in the surface sediments (Table. 1). Higher concentrations of organic matter and photopigment descriptors were detected at vegetated sediments. The highest mean grain sizes were found in unvegetated sediments. Meiofauna density did not differ between bottom types. On the other hand, macrofauna density was much higher at vegetated bottom (Table. 1). The full set of 13 environmental variables described above was tested for collinearity (Draftsman plot and Spearman correlation matrix). Redundant variables with correlations *R* > 0.9 were omitted from the model (only chl *a*/POC and δ^15^N). To determine the best combination of predictor variables, the forward selection procedure was used (for details see Anderson et al. 2008).

**Table 1:** Mean and standard deviation values for environmental parameters measured at the study localities

Environment	Vegetated		Unvegetated		PERMANOVA
	Mean	St. dev.	Mean	St. dev.	Ps-F
POC/PON	8.81	1.61	8.03	1.84	2.8
POC	0.18	0.03	0.12	0.03	45.3***
PON	0.02	0.00	0.02	0.00	14.3***
chl *a*	6.55	2.24	4.54	1.25	5.2*
Mean grain size	1.96	0.12	1.98	0.09	0.4
Sorting	0.52	0.09	0.52	0.08	0
Fine sand	54.19	14.67	50.78	12.76	2.3
Coarse sand	4.07	1.76	5.34	2.85	3.8
δ^13^C	54.19	14.67	50.78	12.76	2.3
δ^15^N	3.38	0.52	1.95	1.05	25.24***
chl%	59.72	16.65	63.34	16.72	0.3
CPE	10.53	2.43	7.74	3.34	8.3**
c/chl	0.06	0.16	0.03	0.01	1
Meiofauna density	1705.41	1019.16	1799.28	1086.48	31.2**
Macrofauna density	30,489.36	5204.25	18,065.96	15,895.13	27.3**
Shoot density	188.22	24.05	0.00	0.00	–
Macrophyte biomass	7.62	2.31	0.00	0.00	–

One-way univariate PERMANOVA results testing differences between vegetated and unvegetated bottom are presented (***P < 0.001; **P < 0.01; *P < 0.05). All details are described in Jankowska et al. 2014

TBNs differed significantly between months (Ps-F 21.7, *P* < 0.001) and bottom types (Ps-F 24.7, *P* < 0.001 PERMANOVA). In both months and at both localities, TBNs were significantly higher at vegetated bottom sites (2.8 ± 1.45 [cell/g sed. DW*10^7^] on average) than at unvegetated bottom sites (1.85 ± 2.05 [cell/g sed. DW*10^7^] on average). For BBM, the same trend was observed (months Ps-F 23.9, *P* < 0.001, bottom types Ps-F 6.7, *P* < 0.01; vegetated: 10.6 ± 5.1 [μgC/g sed. DW]; unvegetated: 8.1 ± 2.4 [μgC/g sed. DW] on average (Fig. 2).

**Fig. 2:**
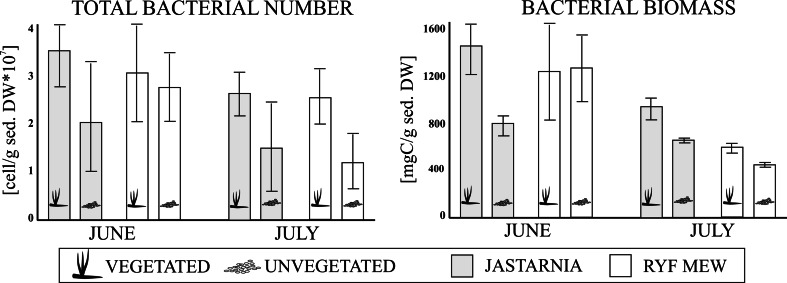
The bacteria characteristics (total bacterial number [cell/g sed. DW*10^7^] and bacterial biomass [μgC/g sed. DW]) recorded in 2 months (June, July), at the two locations (Jastarnia, Ryf Mew) and two bottom types (vegetated, unvegetated)

DISTLM showed significant effects of BT, Lt, shoot density, phytobenthos biomass, coarse sand, meiofauna density (*P* < 0.01), TN, and sorting (*P* < 0.05) on TBNs (Table. 2). Similar sets of variables were significantly correlated with the patterns of BBM: BT, shoot density, phytobenthos biomass, meiofauna, (*P* < 0.01), Lt, TN, chl, and coarse sand (P < 0.05) (Table. 2). For TBNs, meiofauna explained 26.5 %, locality 21.4 %, coarse sand 20.9 %, bottom type 19.6 %, shoot density 18.5 %, phytobenthos biomass 18.6 %, TN, and sorting 14.1 % of the total variation of the number of cells (as indicated by marginal tests). No significant effect of the other ten variables was detected (*P* > 0.05) (Table. 2). For biomass, BT explained 24.2 %, shoot density 24.1 %, phytobenthos biomass 22.9 %, meiofauna density 17.1 %, coarse sand 14.9 %, chl 13.1 %, TN 12.9 %, and Lt 11.8 % of the variability (as indicated by marginal tests). No significant effect of the other ten variables was detected (*P* > 0.05) (Table. 2). For sequential tests, the only significant variables explaining both TBNs and BBM were meiofauna density and bottom type (Table 2) together explaining 48.3 % (TBNs) and 40.5 % (BBM) of variability. Both variables act independently as indicated by the percentage of explained variability—26.5 % (bottom type) and 21.8 % (meiofauna) for TBNs, whereas 24.1 % (bottom type) and 16.4 % (meiofauna) for BBM.

**Table 2:** Results of DISTLM procedure for fitting environmental variables (POC/TN ratio, POC—organic carbon content, TN—total nitrogen content, chl *a*—chlorophyll *a* content, mean grain—mean grain size, fine sand fraction, coarse sand fraction, sorting of sediment, δ^13^C, chl%—percentage of chlorophyll *a* in sediment, CPE—chloroplastic pigments equivalents, meiofauna density (ind. 10 cm^−2^), macrofauna density (ind. m^−2^), seagrass shoot density (shoot no m^−2^), phytobenthos biomass (g dwt^−1^), Mt—month, Lt—location, BT—bottom type) to bacteria univariate characteristics (TBN—total bacterial number [cell/g sed. DW*10^7^], BBM—bacterial biomass [μgC/g sed. DW]). % Var—percentage of explained variance, % Cum—cumulative percentage of variance. Significant effects are indicated (***P < 0.001; **P < 0.01; *P < 0.05). Insignificant effects (*P* > 0.05) are printed in italics

Marginal tests				
Variables	TBN		BBM	
	Ps-F	Var%	Ps-F	Var%
POC/TN	*0.3*	0.8	*0.1*	0.2
POC	*2.8*	7.2	*2.8*	7.2
TN	6.1*	14.1	5.5*	12.9
chl *a*	*3.3*	8.3	5.6*	13.1
Mean grain size	*0.5*	1.4	*0.8*	2.2
Fine sand	*2.6*	6.5	*1.9*	5.0
Coarse sand	9.8**	20.9	8.1*	14.9
Sorting	6.1*	14.1	*3.7*	9.1
δ^13^C	*2.6*	6.5	*1.9*	5.0
chl%	*0.1*	0.2	*0.1*	0.2
CPE	*2.7*	6.7	*3.8*	9.3
Meiofauna density	13.3**	26.5	7.6**	17.1
Macrofauna density	*0.1*	0.0	*0.1*	0.1
Shoot density	8.4**	18.5	11.7**	24.1
Phytobenthos biomass	8.4**	18.6	11.0**	22.9
M	*0.7*	1.2	*1.2*	3.1
Lt	10.1**	21.4	4.9*	11.8
BT	9.0**	19.6	11.8**	24.2
Sequential tests				
	TBN			
Sequential tests	Ps-F	Var%	Cum%	
Meiofauna	13.3**	26.5	26.5	
BT	*12.2*	21.8	48.3	
	BBM			
Sequential tests	Ps-F	Var%	Cum%	
BT	11.8**	24.1	24.2	
Meiofauna	10.0**	16.4	40.5	

This is the first study in the Baltic Sea documenting the increase of bacteria abundance and biomass within the seagrass vegetated sediments as compared to the bare sands. There have been few studies dealing with bacteria inhabiting vegetated sediments in other regions (Danovaro 1996; James et al. 2006; García-Martínez et al. 2008). The increase of biomass of the benthic microbial community within seagrass meadows was documented by Danovaro et al. (1994) who found higher TBNs and BBM in the sediment covered by *P. oceanica* as compared to unvegetated bottom. Moreover, for the *P. oceanica* meadows in the Mediterranean Sea, there is also an evidence of a very high bacterial diversity (García-Martínez et al. 2008). García-Martínez et al. (2008) indicated that a decline of the seagrass meadow causes the decline of the microbial community. Previous studies that aimed to identify factors controlling bacterial communities in coastal sediments documented a relationship between bacterial community respiration and microalgal primary production or biomass (Hargrave 1969) as well as between TBNs and phytoplankton activity (Fuhrman and Azam 1980). Danovaro (1996) stated that the correlation between BBM and chlorophyll *a* indicated bacteria response to changes in the microphytobenthos biomass and production (Van Wambeke et al. 1984; Cammen and Walker 1986). One of the two most important drivers responsible for bacterial abundances indicated in the present study is the bottom type so the presence or absence of eelgrass meadows. As measured values of the bacteria community recorded at the vegetated seabed are much higher than those of the bare bottom, seagrass meadows cause therefore a significant increase in the measured bacteria parameters (TBN, BBM). Positive effects of seagrass meadows on bacteria are direct by creating a substrate for bacteria and indirect by enhancing the organic matter content of the sediment as well as by improving sediment conditions. The quantity and quality of organic matter as well as the sediment type are important drivers explaining bacteria community structure (Danovaro and Fabiano 1995). Therefore, the main reason for higher TBN and BBM at vegetated bottom may be a higher concentration of nutrients and food sources (detrital organic matter). The entrapment of organic matter (indicated by increased of POC and TN concentrations in sediments) at vegetated bottom was indeed observed in the studied sites as well as TBNs and BBM were significantly correlated to the TN and chlorophyll *a* concentrations in the surface sediments (Table 2). Chlorophyll *a* and pheopigment concentrations in sediment are often treated as a proxy of microalgal production, both planktonic and benthic (Danovaro and Fabiano 1995). In the present study, a higher concentration of photosynthetic pigments was reported for the vegetated bottom which may be explained by sediment stabilization by vegetated structures and thus reduction of water flow that cause enhancement of microalgal production. The recycling of the carbon derived from microphytobenthos by bacteria was recently described by Oakes et al. (2012), and the relatively high POC/TN values (ten on averages) can indicate strong decomposition processes caused by bacteria inhabiting seagrass meadows (Danovaro and Fabiano 1995). Therefore, increased grazing of bacteria on unlimited resources (microphytobenthos) may be an important driver responsible for bacterial grows at vegetated bottom.

In the present study, meiofauna was showed by DISTLM analyses to be the second (next to bottom type) variable best correlated to bacterial characteristics. What is more the effects of those two factors—bottom type and meiofauna densities, were not overlapping so act independently as indicated by the significant effects for both factors reported in sequential test. Indeed, the effects of vegetation and meiofaunal consumers are of different natures. Canon et al. (1998) suggested that besides the food/nutrients availability, the bacterial standing stocks in marine sediments are largely top-down controlled by predation pressure. Most authors underline the significant effects of meiofauna and little or no effects of macrofauna on bacteria (e.g., Papadimitriou et al. 2005). The grazing of meiofauna on bacteria was documented as a factor controlling bacterial abundance only when their densities were high (Alberetelli et al. 1999). Recent mesocosm experiment (Bonaglia et al. 2014) indicates that nematodes, which are the predominant meiofauna components in marine sediments, stimulate nitrifiers and denitrifiers by secretion of nitrogen. Current experiments show specific interactions between some meiofauna species and bacteria. One example is that harpacticoid copepods are gardening microorganisms growing on their fecal pellets (De Troch et al. 2005). In the Puck Bay sediments, the relationship between the two groups of organisms is positive (as indicated by Spearman correlation). That may indicate a bottom-up control by which higher bacterial standing stocks result in better food conditions and thus in a higher number of meiofaunal consumers. However, these interactions may operate in both directions, as some studies reported that intensive grazing by meiofauna may induce intensification in bacterial production and thus an increase of microorganism abundance (Bonaglia et al. 2014).

The values of TBNs documented for the Puck Bay sediments were lower than those recorded in other regions (present study—3.6 [cell/g sed. DW*10^7^], *P. oceanica* in the Liguarian Sea—5.8 [cell/g sed. DW*10^8^] (Danovaro et al 1994), *Zostera noltii* in the Wadden Sea—32 [cell/g sed. DW*10^8^] (Llobet-Brossa et al. 1998), *P. oceanica* in the Adriatic Sea—13.8 [cell/g sed. DW*10^8^] (Danovaro and Gambi 2002). These differences could be explained by relatively low seagrass density and biomass in the study area as compared to other regions (Jankowska et al. 2014). The temperature does not seem to be important as it was not controlling bacterial abundances in seasonal study in the Mediterranean Sea (Danovaro et al. 1994, 1998). Seagrass meadows in the Puck Bay remain in the state of natural recovery and are expanding (http://water.iopan.gda.pl/projects/Zostera/index-pl.html). Therefore, the effects of *Z. marina* environmental engineer effects on benthic system can increase accordingly.

The strong positive effect of seagrass meadows on bacteria abundance reported by this study highlights the importance of the macrophyte vegetation for the microbial communities associated with marine sediments. More studies focusing on the microbial components of seagrass meadow biocenosis are needed to improve our understanding of the functioning of this system and its importance in the coastal areas.
